# Contribution of Chronic Disease to the Burden of Disability

**DOI:** 10.1371/journal.pone.0025325

**Published:** 2011-09-22

**Authors:** Bart Klijs, Wilma J. Nusselder, Caspar W. Looman, Johan P. Mackenbach

**Affiliations:** Department of Public Health, Erasmus MC, University Medical Centre, Rotterdam, The Netherlands; Finnish Institute of Occupational Health, Finland

## Abstract

**Background:**

Population ageing is expected to lead to strong increases in the number of persons with one or more disabilities, which may result in substantial declines in the quality of life. To reduce the burden of disability and to prevent concomitant declines in the quality of life, one of the first steps is to establish which diseases contribute most to the burden. Therefore, this paper aims to determine the contribution of specific diseases to the prevalence of disability and to years lived with disability, and to assess whether large contributions are due to a high disease prevalence or a high disabling impact.

**Methodology/Principal Findings:**

Data from the Dutch POLS-survey (Permanent Onderzoek Leefsituatie, 2001–2007) were analyzed. Using additive regression and accounting for co-morbidity, the disabling impact of selected chronic diseases was calculated, and the prevalence and years lived with ADL and mobility disabilities were partitioned into contributions of specific disease. Musculoskeletal and cardiovascular disease contributed most to the burden of disability, but chronic non-specific lung disease (males) and diabetes (females) also contributed much. Within the musculoskeletal and cardiovascular disease groups, back pain, peripheral vascular disease and stroke contributed particularly by their high disabling impact. Arthritis and heart disease were less disabling but contributed substantially because of their high prevalence. The disabling impact of diseases was particularly high among persons older than 80.

**Conclusions/Significance:**

To reduce the burden of disability, the extent diseases such as back pain, peripheral vascular disease and stroke lead to disability should be reduced, particularly among the oldest old. But also moderately disabling diseases with a high prevalence, such as arthritis and heart disease, should be targeted.

## Introduction

Population ageing is expected to lead to a sharp increase in the occurrence of disability. Disability is associated with an increased need for social services, e.g. healthcare, and a loss in quality of life [1], [2], [3]. To enable the future health care system to cope with increasing demands, and to avoid strong decrements in the quality of life, it is crucial to develop strategies that effectively lead to reductions in the burden of disability. One of the first steps crucial in developing these strategies is to identify which diseases contribute most to the total burden of disability, but also to clarify whether large contributions are related with a high prevalence of disease or with a high disabling impact, i.e. a high extent the disease leads to disability.

To date, only a limited number of studies have assessed the contribution of specific diseases to the burden of disability [4], [5], [6], [7], [8], [9], [10], [11], [12], [13], [14]. Unfortunately, most of these studies were based on relatively old data [4], [5], [6], [7], [8], [9]. As both the prevalence of chronic diseases and their disabling impacts change over time, the results of these studies may be outdated [13], [14]. Furthermore, only two studies explicitly addressed the role of disease prevalence and disabling impact in contributions to disability [5], [6]. The most comprehensive study assessing burden of disease has probably been WHOs Global Burden of Disease study (GBD) [12]. In this study, however, disease burden is quantified on the basis of panel valuations of health states rather than actual presence of physical or mental disabilities [12].

Most of the previous studies were based on cause elimination techniques, which provide outcome measures that reflect the reduction in the prevalence of disability if the disease would no longer be present [4], [7], [8], [11]. Drawbacks of this method are that the results may be inconsistent in a situation of co-morbidity, as they depend on the ordering of the elimination, and that contributions of all diseases do not add up to the total disability prevalence. Recently, Nusselder et al. developed a methodology, based on an additive regression technique, that enables exact partitioning of the burden of disability into additive contributions of disease in the presence of co-morbidity [5], [15].

The aim of this study was to investigate the current contribution of specific chronic diseases to the total burden of disability in mobility and activities of daily living among the elderly, both in terms of disability prevalence and years lived with disability. This study is among the first to highlight the role of both the prevalence and the disabling impact of specific diseases to determine their contribution to the burden of disability.

## Methods

### Study population

The study population consisted of subjects from seven successive years (2001–2007) of the POLS health and labor survey, which is being conducted by Statistics Netherlands. The survey does not include the institutionalized population. To account for selective non-response and to ensure representativeness for the Dutch non-institutionalized population, weights were used that were attached to the data.

**Figure 1:**
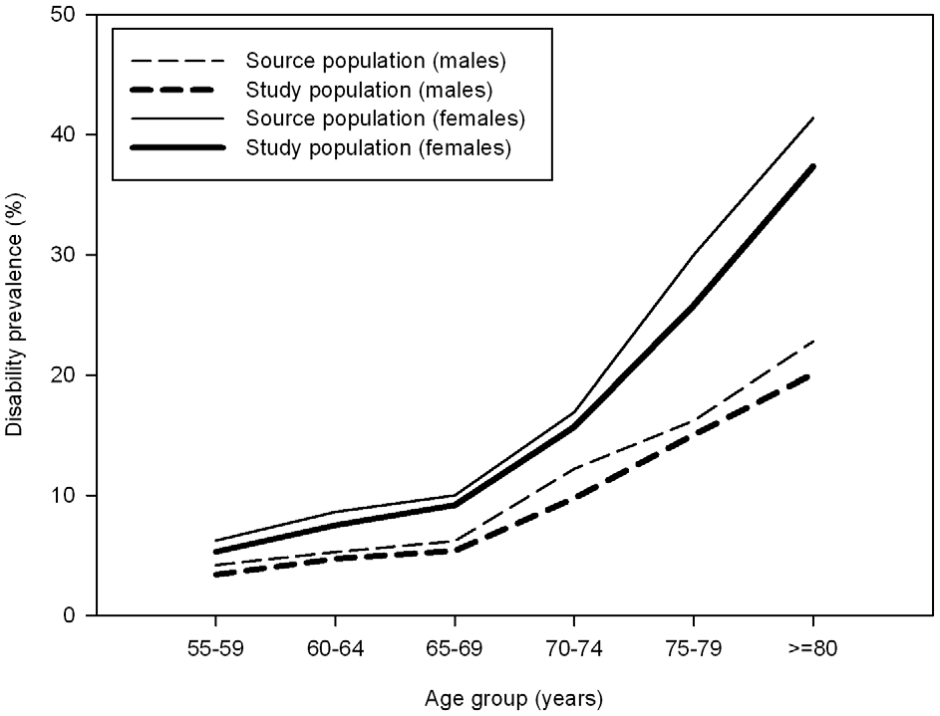
Prevalence of disability by age. The source population consisted of all respondents to the POLS health and labor survey, the Netherlands, 2001–2007, aged 55 and older (n = 17,404). The study population equals the source population minus all subjects who had information missing on the presence of diseases (n = 13,635).

Information on disabilities in mobility and activities of daily living was collected through face-to-face interviews and information on the presence of chronic disease through written questionnaires. From 2001–2007, 110,766 subjects were approached and the response was 62%. For our analyses selected elderly subjects who were 55 years and older (n = 17,404) were selected. 22% of these subjects could not be included in the study population because they lacked disease information. Table 1 provides further detailed information on numbers of persons in the study population and Figure 1 on the difference between the source and study population.

**Table 1:** Numbers of subjects in the study sample.

	males			females		
	55–64	65–79	> = 80	55–64	65–79	> = 80
total	3412	2873	478	3187	3005	680
elementary education	531	695	149	680	1160	359
secondary education	1928	1499	232	2003	1546	265
tertiary education	932	656	95	495	286	52
education missing	21	23	2	9	13	4
DM	262	365	55	168	353	97
stroke	130	233	50	88	159	53
heart disease	330	599	116	107	297	100
PVD	119	207	50	72	183	67
cancer	130	278	66	244	306	80
CNSLD	229	283	61	239	314	67
backpain	414	258	46	380	407	102
arthritis	580	629	147	868	1254	358
disorder neck/arm	432	316	46	623	577	127
other	600	487	118	918	906	269
no disease reported	1620	1014	119	1283	845	131
ADL disabled	137	263	96	200	472	254

Abbreviations: CNSLD  =  chronic non-specific lung disease; DM  =  diabetes mellitus; PVD  =  peripheral vascular disease (upper extremity excluded).

Numbers of persons with diseases do not add up to total because of co-existence of diseases.

### Disability

Subjects were asked if they were able to ‘walk up and down the stairs’, ‘walk outside’, ‘enter/leave the house’, ‘sit down/get up from a chair’, ‘move around on the same floor’, ‘get in/out of bed’, ‘eat/drink’, ‘get dressed/undressed’, ‘wash face/hands’ and ‘wash completely’ and could answer with ‘without difficulty’, ‘with minor difficulty’, ‘with major difficulty’ and ‘only with help’. Someone was considered disabled if he or she opted for one of the latter two answers at least once.

### Definition of disease groups

Information on the presence of a range of diseases was collected in the questionnaire. From the original questions ‘cardiovascular disease’ (CVD) was compiled, containing ‘stroke’, ‘heart disease’ and ‘peripheral vascular disease (PVD)’, and ‘musculoskeletal disease’ was compiled, containing ‘back pain’, ‘arthritis’ and ‘disorder neck/arm’. Furthermore, ‘diabetes mellitus (DM)’, ‘cancer’, ‘chronic non-specific lung disease (CNSLD)’ and ‘other’ were distinguished. Appendix S1 shows the original questions and the diseases that were distinguished. Skin cancer was not included because it is not associated with disability. PVD did not include vascular disease of the upper extremity. In subjects who had suffered from back pain or disorders of the neck/arm but indicated that they currently did not suffer anymore, the condition was regarded as no longer present. If a disease was defined on the basis of multiple questions and information on any of the questions was missing while none of the questions indicated the presence of a disease, the disease information was considered missing.

### Statistical methods

Disability prevalence by cause was estimated from individual information on the presence or absence of disability, the presence or absence of the selected disease groups, gender and age. A method based on a multivariate additive regression model was used, which is described in more detail elsewhere [5], [6], [16]. This method takes into account that persons who do not report a disease may be disabled (this risk is referred to as “background”) and that persons can have more than one disease (co-morbidity). Disability in persons without a reported disease is entirely attributed to background. Disability in persons with at least one disease is attributed partly to background and partly to the disease(s). We assume that causes of disability (diseases and background risk) act as independently competing causes. Assuming independence, the hazard of someone having disease A and B (and zero background risk) equals hazard A + hazard B. In a situation of no competing risk (one disease and zero background risk) the hazard can be easily converted to a probability of being disabled (1-exp(- disease hazard)). When more causes are competing, the probability of being disabled from the specific disease is lower than in the situation of no competition. In the footnote of table 2, a calculation example is given of how was dealt with co-morbidity.

**Table 2:** Prevalences and disabling impacts of disease.

	disease prevalences (%)			disabling impacts of disease (hazard)		
	55–64	65–79	> = 80	55–64	65–79	> = 80
*males*						
DM	7.7 (6.9–8.7)	12.6 (11.5–13.9)	10.8 (1.6–9.0)	0.01 (0.00–0.02)	0.02 (0.00–0.05)	0.03 (0.00–0.08)
stroke	3.8 (3.2–4.4)	8.1 (7.2–9.1)	10.9 (8.4–14.1)	0.08 (0.05–0.12)	0.16 (0.10–0.23)	0.28 (0.17–0.42)
heart disease	9.6 (8.7–10.6)	20.9 (19.4–22.4)	23.3 (19.8–27.2)	0.01 (0.00–0.03)	0.03 (0.01–0.06)	0.05 (0.01–0.10)
PVD	3.4 (2.9–4.1)	6.9 (6.1–7.9)	10.6 (8.1–13.7)	0.09 (0.05–0.13)	0.17 (0.10–0.26)	0.30 (0.17–0.47)
cancer	3.7 (3.1–4.4)	9.7 (8.7–10.9)	14.3 (11.4–17.8)	0.01 (0.00–0.03)	0.02 (0.00–0.05)	0.04 (0.00–0.09)
CNSLD	6.6 (5.8–7.5)	10.2 (9.1–11.4)	12.6 (9.9–15.9)	0.08 (0.05–0.11)	0.15 (0.10–0.20)	0.26 (0.16–0.38)
backpain	11.9 (10.9–13.0)	8.7 (7.7–9.8)	8.9 (6.7–11.7)	0.08 (0.05–0.11)	0.16 (0.11–0.22)	0.29 (0.17–0.42)
arthritis	16.7 (15.5–18.0)	21.9 (20.4–23.5)	31.0 (27.0–35.3)	0.03 (0.02–0.05)	0.07 (0.04–0.09)	0.12 (0.06–0.19)
disorder neck/arm	12.7 (11.6–13.8)	10.9 (9.8–12.1)	9.5 (7.2–12.4)	0.00 (0.00–0.02)	0.01 (0.00–0.04)	0.01 (0.00–0.08)
other	17.3 (16.1–18.6)	17.4 (16.0–18.8)	25.1 (21.4–29.2)	0.03 (0.01–0.04)	0.05 (0.02–0.08)	0.09 (0.04–0.15)
no disease reported	48.1 (46.4–49.8)	35.4 (33.6–37.1)	25.8 (22.1–30.0)			
*females*						
DM	5.4 (4.7–6.2)	11.7 (10.6–12.9)	14.7 (5.9–16.0)	0.07 (0.04–0.10)	0.11 (0.06–0.16)	0.23 (0.13–0.37)
stroke	2.6 (2.1–3.2)	5.2 (4.5–6.1)	7.9 (6.1–10.2)	0.10 (0.05–0.16)	0.16 (0.08–0.24)	0.35 (0.18–0.55)
heart disease	3.2 (2.7–3.9)	9.9 (8.9–11.0)	14.4 (11.9–17.2)	0.06 (0.03–0.10)	0.10 (0.05–0.15)	0.22 (0.11–0.36)
PVD	2.3 (1.8–2.8)	6.4 (5.6–7.4)	9.9 (7.9–12.4)	0.11 (0.06–0.18)	0.17 (0.09–0.27)	0.38 (0.21–0.60)
cancer	7.6 (6.7–8.5)	10.2 (9.2–11.4)	11.5 (9.3–14.0)	0.01 (0.00–0.03)	0.01 (0.00–0.04)	0.03 (0.00–0.09)
CNSLD	7.4 (6.6–8.4)	10.5 (9.4–11.6)	10.2 (8.1–12.8)	0.06 (0.03–0.10)	0.10 (0.05–0.15)	0.22 (0.10–0.34)
backpain	12.1 (11.0–13.3)	13.7 (12.5–15.0)	15.3 (12.7–18.2)	0.12 (0.09–0.16)	0.20 (0.14–0.26)	0.44 (0.30–0.60)
arthritis	27.1 (25.6–28.7)	41.6 (39.9–43.4)	53.6 (49.9–57.3)	0.07 (0.05–0.09)	0.11 (0.08–0.14)	0.24 (0.17–0.32)
disorder neck/arm	19.8 (18.4–21.2)	19.4 (18.0–20.8)	19.7 (16.8–22.9)	0.03 (0.01–0.05)	0.04 (0.01–0.07)	0.09 (0.03–0.17)
other	29.3 (27.7–30.9)	30.4 (28.8–32.1)	39.0 (35.4–42.7)	0.04 (0.02–0.05)	0.06 (0.03–0.09)	0.13 (0.07–0.20)
no disease reported	40.0 (38.3–41.7)	27.9 (26.4–29.6)	19.7 (16.8–22.8)			

Abbreviations: CNSLD  =  chronic non-specific lung disease; DM  =  diabetes mellitus; PVD  =  peripheral vascular disease (upper extremity excluded).

5 year age groups were used to calculate the disabling impact of diseases and the prevalence of disability by cause (table 3). To summarize and as is shown in the table, disabling impacts were also calculated using three age-aggregated groups (55–64.9, 65–79.9, > = 80). ‘Background’, representing presence of disability irrespective of disease presence, is preferably modelled according to 5 year age groups and could therefore not be shown in the table.

The disabling impact represents the rate of disability from a specific cause given that the disease is present. Adding these specific disability rates for the diseases present and the background rate of disability (by age and gender) gives the total disability rate for a specific exposure group. The proportion of the cause-specific rate in total rate is used to divide the probability of disability in this group by cause. For example, for males aged 75–79 with PVD and arthritis, adding the background rate (0.03), the rate for PVD (0.17), and the rate for arthritis (0.07) yields a total disability rate of 0.27 and a total probability of disability of 0.24 (1 − exp(−0.27)  = 0.24). The probability of disability from background in this group is 0.03 (0.03/0.27 * 0.24), that of PVD is 0.15 (0.17/0.27 * 0.24), and that of arthritis is 0.06 (0.07/0.27 * 0.24).

The disabling impact for males and females was significantly different (*P*<0.05) for DM, heart disease, cancer, arthritis and disorder neck/arm.

To estimate cause-specific disability prevalence from cross-sectional data the following assumptions were made. First, the distribution of disability by cause is explained entirely by diseases that are (still) present at the time of the survey and the risk of disability in absence of any reported diseases. Second, this distribution is proportional to the distribution of the risk of becoming disabled in the time-period preceding the survey. Thirdly, causes of disability (diseases and background) act as independently competing causes.

The regression model is specified as follows:
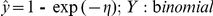


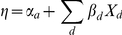
where 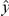 is the estimated probability that the person has disability, e is the base of the natural logarithm and 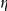 the linear predictor. The latter is defined as the sum of the background rate by age (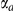) and the cause-specific rates of disability (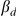, labeled as “disabling impact”) for the disease groups (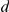) that are present in the respondent (given by the dummy variables 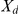). Background was handled as a cause that is prevalent for everyone; the rate of background is age dependent (5-year age groups). The disabling impact 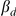 may also vary by age. As the full age-interaction term would require 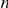 (number of age classes) times 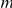 (number of diseases) different parameters, the rank of the interaction was reduced to one, which means that the age-specific disabling impact of each disease, 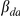, is estimated as the product of an age pattern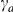 which is equal for each disease, and a disease effect 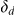, which varies by disease, but not by age (Reduced Rank Regression) [17], [18]. While a one rank solution restricts the age pattern to be the same for all diseases, also second rank solutions were fitted (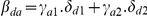) and scaled deviances were compared to test for differences in age patterns for different diseases. Adding a one rank interaction improved the fit of the model (log-likelihood ratio test with *P*-value of 0.05), indicating that the disabling impact varies by age. Because adding a second rank did not further improve the fit of the model, the same age pattern for all diseases was used. Models were fitted using a quasi-Newton method that was programmed for the statistical package R version 2.7.1. [19]. All analyses were done seperately for men and women, as the log-likelihood ratio test indicated that both the rates of background and the disease-specific rates given sex-specific rates of background differed signifcantly by sex. The significance of the differences in the disabling impact between males and females was assessed by assuming normal distribution of the parameters with the mean of the original ones as the standard error.

### Calculation of number of disabled by cause across subgroups

Disability prevalence by cause depends on the prevalence of the disease (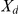) and the disabling impact of the disease (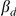 or 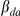). Analogous to using the proportional distribution of mortality rates to obtain probabilities of death in the presence of competing causes (so called “crude probabilities”), the attribution of disease 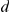 is 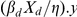 and of background is 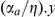 [5], [6]. Applying these formulas gives for every individual the probability of being disabled caused by background *or* disease (if present). Adding the cause-specific probabilities of an individual gives the probability of being disabled for that individual. Adding the cause-specific probabilities of all persons in the dataset, or in a specific age group, gives the total number of disabled by cause in the population, or in that age group. Dividing the number of disabled persons by cause by the total number of persons gives the proportion of disability by cause.

Years lived with disability at age 55 by cause were obtained using the Sullivan method [20]. The Sullivan method uses the prevalence of disability in each age group to divide the number of person-years into years lived with and without disability [20]. Instead of using the age and sex specific prevalence of disability, we used age, sex and cause specific prevalence of disability, yielding the years with disability by each cause. Adding the years with disability by each cause yields the life expectancy with disability, as the sum of the cause-specific disability add to the total prevalence. A life table for the Dutch population 2001–2007, available from the EHEMU database, was used [21].

Confidence intervals around the estimates of disabling impacts, prevalences of disability by cause and contributions to LED at age 55 were obtained with bootstrapping based on 1000 replicas [22]. For the disabling impacts and prevalences by cause we used non-parametric bootstrapping. For the bootstrap of life expectancy, which is used in combination with prevalence by cause to calculate LED, we used parameterized bootstrap, assuming Poisson distribution of the numbers of deaths.

The software for additive regression and for calculation of disability prevalence by cause is available from the authors on request.

## Results

In males, the prevalence of disability increased from 4% at ages 55–59 to 20% at ages older than 80. In females, this was from 6% to 37% (Figure 1). Also persons without any disease reported disability.

The contribution of diseases to the prevalence of disability depends both on the prevalence and disabling impact of disease. Among males, arthritis, heart disease and other, and at younger ages also back pain, had the highest prevalences (Table 2). Among females these were arthritis, other, disorder neck/arm and back pain, and at older ages also DM and heart disease.

Among males, PVD had the highest disabling impact (Table 2). Furthermore, stroke, CNSLD and back pain showed high disability risks. Among females, back pain had the highest disabling impact, but stroke and PVD also showed high impacts. In both sexes, cancer did not lead to much disability, as was the case for DM and heart disease in males. The disabling impacts of DM, heart disease, arthritis and disorder neck/arm were significantly higher among females than among males (p<0.05). Although the disabling impact of cancer was low, it was significantly higher among males. The disabling impact of the diseases increased with age (*P*<0.05).

**Figure 2:**
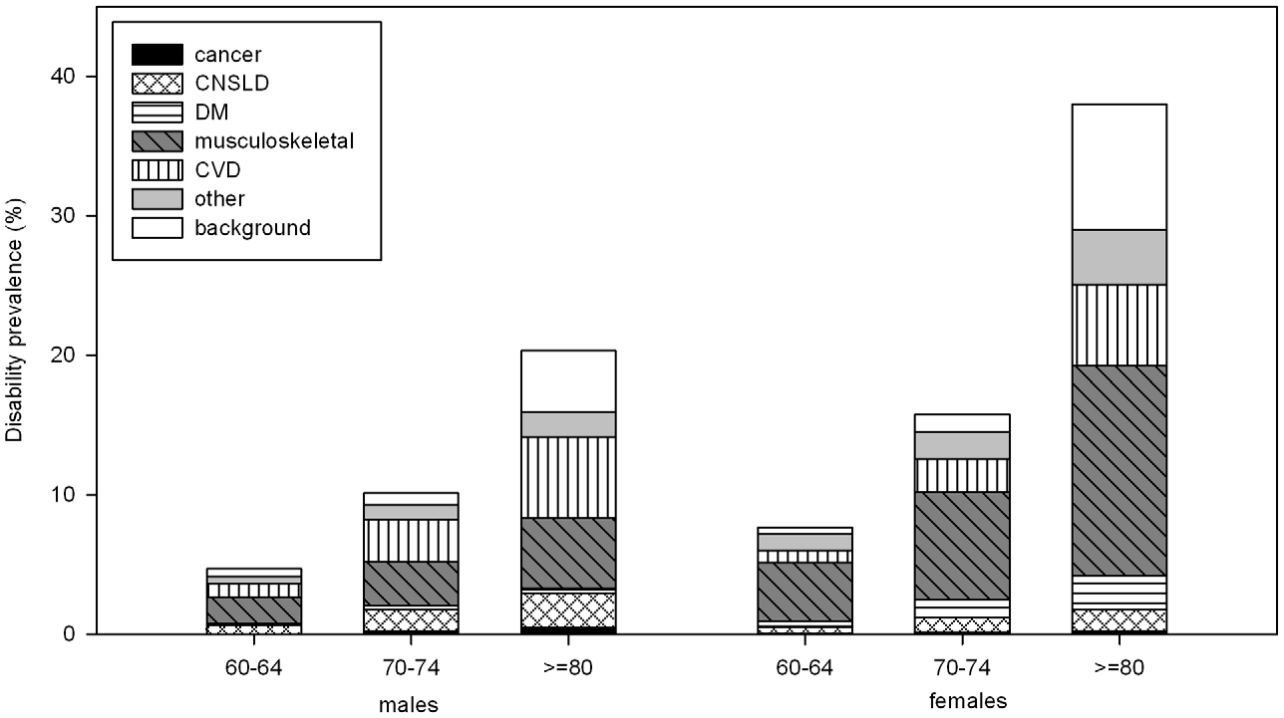
Prevalence of disability by cause. Abbreviations: CNSLD  =  chronic non-specific lung disease; CVD  =  cardiovascular disease; DM  =  diabetes mellitus; PVD  =  peripheral vascular disease (upper extremity excluded). Contributions of specific diseases to the prevalence of disability were estimated on the basis of diseases prevalence and disabling impact in the study sample from the POLS health and labor survey, the Netherlands, 2001–2007. The disabling impact represents the rate of disability from a specific cause given that the disease is present. Adding specific disability rates for the diseases present and the background rate of disability (by age and gender) gives the total disability rate for a specific exposure group. The contributions of specific diseases presented in the figure add up to the total prevalence of disability.

In males, the most important contributors to the prevalence of disability were musculoskeletal disease and CVD (Table 3, Figure 2). Musculoskeletal disease accounted for 40% of the prevalence of disability below age 65, which was about twice the contribution of CVD. At older ages the two conditions contributed equally. Most of the disability attributed to CVD was caused by stroke and PVD. In males younger than 65, most disability attributed to musculoskeletal disease was caused by back pain. At older ages, the largest part was caused by arthritis. CNSLD contributed less than musculoskeletal disease and CVD, but was still responsible for 10–15%. In females, musculoskeletal disease was by far the most important contributor and accounted for 40–50% of the disability burden. Disability attributed to musculoskeletal disease was mostly caused by arthritis, but back pain was also an important cause. The second important cause was CVD, contributing more than 15% in females aged 80 and older. Stroke, heart disease and PVD all three contributed importantly to the disability attributed to CVD. DM was also important and contributed 5–8%.

**Table 3:** Contributions of disease to prevalence of disability and to life expectancy with disability at age 55.

	contribution to prevalence of disability (% points)			contribution to LED at age 55 (years)
	55–64	65–79	> = 80	
*males*				
DM	0.08 (0.00–0.19)	0.24 (0.00–0.55)	0.33 (0.00–0.77)	0.06 (0.00–0.14)
stroke	0.28 (0.17–0.42)	1.14 (0.73–1.58)	2.27 (1.37–3.26)	0.34 (0.23–0.46)
heart disease	0.16 (0.06–0.30)	0.66 (0.27–1.15)	1.17 (0.46–2.02)	0.18 (0.08–0.31)
PVD	0.28 (0.15–0.42)	1.07 (0.63–1.51)	2.34 (1.33–3.38)	0.33 (0.20–0.46)
cancer	0.04 (0.00–0.09)	0.19 (0.00–0.42)	0.44 (0.00–1.08)	0.06 (0.00–0.13)
CNSLD	0.45 (0.30–0.66)	1.35 (0.92–1.84)	2.49 (1.49–3.63)	0.40 (0.27–0.53)
back pain	0.92 (0.62–1.26)	1.25 (0.84–1.72)	1.91 (1.09–2.86)	0.39 (0.27–0.52)
arthritis	0.55 (0.31–0.81)	1.41 (0.83–1.92)	3.07 (1.59–4.74)	0.45 (0.26–0.62)
disorder neck/arm	0.04 (0.00–0.23)	0.07 (0.00–0.38)	0.09 (0.00–0.60)	0.02 (0.00–0.11)
other	0.41 (0.19–0.67)	0.82 (0.38–1.31)	1.82 (1.82–1.82)	0.27 (0.12–0.45)
back ground	0.73 (0.28–1.20)	0.97 (0.43–1.61)	4.37 (1.36–7.91)	0.51 (0.29–0.77)
total	3.93 (3.33–4.62)	9.07 (8.08–10.18)	20.37 (16.98–24.24)	3.01 (2.68–3.31)
*females*				
DM	0.33 (0.18–0.51)	1.14 (0.62–1.66)	2.45 (1.30–3.78)	0.44 (0.24–0.65)
stroke	0.21 (0.10–0.35)	0.69 (0.37–1.08)	1.65 (0.89–2.46)	0.28 (0.15–0.42)
heart disease	0.17 (0.08–0.28)	0.85 (0.41–1.30)	2.04 (0.90–3.18)	0.34 (0.16–0.52)
PVD	0.19 (0.08–0.31)	0.86 (0.41–1.30)	2.12 (1.08–3.27)	0.35 (0.18–0.52)
cancer	0.05 (0.00–0.19)	0.11 (0.00–0.41)	0.22 (0.00–0.83)	0.04 (0.00–0.16)
CNSLD	0.42 (0.21–0.63)	0.93 (0.47–1.43)	1.52 (0.77–2.44)	0.32 (0.17–0.48)
back pain	1.30 (0.94–1.74)	2.30 (1.70–2.94)	4.07 (2.92–5.44)	0.86 (0.64–1.08)
arthritis	1.72 (1.27–2.17)	4.35 (3.36–5.25)	9.72 (7.27–12.17)	1.75 (1.36–2.11)
disorder neck/arm	0.46 (0.13–0.84)	0.72 (0.21–1.28)	1.25 (0.35–2.33)	0.27 (0.07–0.48)
other	1.06 (0.66–1.49)	1.78 (1.13–2.58)	3.97 (2.38–5.94)	0.75 (0.46–1.09)
back ground	0.61 (0.21–1.07)	2.04 (1.21–3.00)	8.96 (5.18–13.35)	1.23 (0.82–1.72)
total	6.48 (5.66–7.40)	15.65 (14.39–16.99)	38.07 (34.48–41.80)	6.64 (6.20–7.06)

Abbreviations: CNSLD  =  chronic non-specific lung disease; DM  =  diabetes mellitus; PVD  =  peripheral vascular disease (upper extremity excluded).

**Figure 3:**
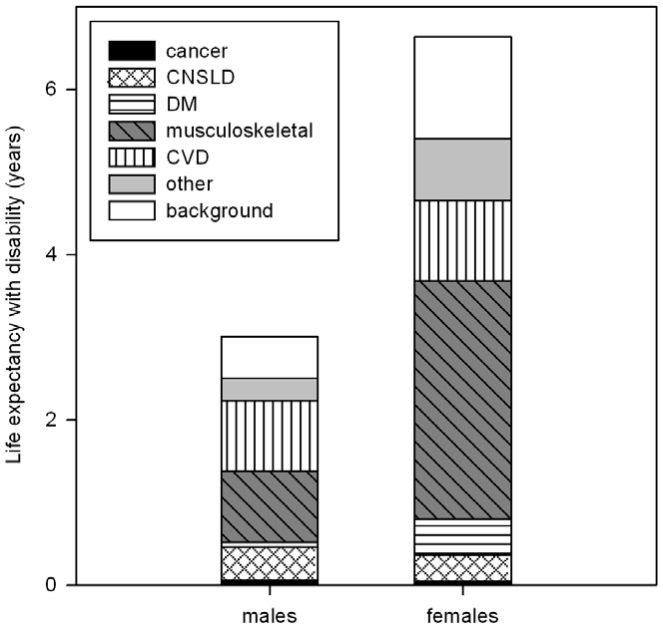
Life expectancy with disability at age 55 by cause. Abbreviations: CNSLD  =  chronic non-specific lung disease; CVD  =  cardiovascular disease; DM  =  diabetes mellitus; PVD  =  peripheral vascular disease (upper extremity excluded). Contributions of specific diseases to the life expectancy with disability were estimated on the basis of estimated contributions of specific disease to the prevalence of disability in the study sample from the POLS health and labor survey, the Netherlands, 2001–2007, in combination with life table information for the Dutch population 2001–2007, available from the EHEMU database. Methods of decomposition are described elsewhere (5).

Diseases contributions to years lived with disability were similar to contributions to the prevalence of disability (Table 3, Figure 3). Of the 3.01 years with disability in males, 0.85 were contributed by CVD and 0.86 by musculoskeletal disease. CNSLD contributed 0.40 years. In females, musculoskeletal diseases were responsible for 2.93 of the 6.64 life years with disability. The contribution of arthritis alone (1.75 years) was about 0.8 years more than the contribution of all CVDs together (0.97 years). DM contributed 0.44 years.

## Discussion

This study is among the first to investigate contributions of various chronic diseases to the prevalence of disability and is the first to present years lived with disability by cause. Musculoskeletal disease is the main contributor and CVD, particularly important among males, is a second. CNSLD is the third contributor among males and DM among females. Within the group of musculoskeletal disease, arthritis-disorder neck arm contributes mostly by a high prevalence and back pain by a high disabling impact, although the prevalence of this condition is also high. Within the group of CVD, heart disease contributes mostly by its high prevalence and PVD and stroke by its high disabling impact. The disabling impact of all diseases increases with age.

### Evaluation of data and methods/limitations

Selection bias may limit the external validity of the results. Possible selection bias caused by non-response (38%) was minimized by using individual weights to adjust for selection effects by age, gender, marital status, urbanization grade, province, employment, health- and smoking status [23].

Twenty two percent of all subjects aged 55 and older lacked disease information and were excluded from the analysis, which led to a slight underestimation of disability in our study (Figure 1). The higher prevalence of disability among non-responders may suggest a slight underestimation of the prevalence and/or disabling impact of some diseases, and hence, that our results should be regarded as conservative.

Our results may not be generalizable to the institutionalized population, which has a higher prevalence of disability and also the relative contribution of specific diseases to the total burden of disability inside institutions may differ from our estimates [24], [25]. However, in the Netherlands only a minor part of elderly people lives in an institution (i.e. 90% of those aged 80–85 still live at home), hence, bias due to excluding the institutionalized population is probably small and negligible at younger ages [25].

Due to differences in the extent diseases have remained undiagnosed in the population and due to differences in the reference periods used in the questionnaire, some variation may exist in the extent the prevalences derived from the POLS survey reflect true prevalences. Previous literature showed that self-report of most chronic conditions is fairly accurate, except for arthritis, which may be underestimated as well as overestimated [26]. If the prevalence of arthritis or another disease in our study was underestimated, subjects with less severe forms of the disease would be most likely to be uncounted and, hence, the disabling impact would be overestimated. This implies that although there may be some bias in the estimates of disease prevalence, this is nullified by a bias in opposite direction in the disabling impact. Hence, the bias in the estimates of contributions of specific diseases to the prevalence of disability and life expectancy with disability is likely to have remained small. Self-report of ADL disability has been found to correlate well with performance based measures and, hence, is expected not to have affected the results substantially [27].

Using a less stringent definition of disability, defined as one or more items answered with “at least minor difficulty” resulted in a lower percentage of disability explained by the diseases included. Our substantive conclusions regarding which diseases contributed most remained unaffected. Due to a lack of power, using a more stringent cut-level could not be evaluated.

The cross-sectional nature our methods and data did not allow identifying cases in which disability was present prior to the onset of the disease. In these cases the disability might be falsely attributed to the disease.

It was decided not to exclude conditions such as ‘dizziness with falling’ and ‘involuntary loss of urine’, which have an intermediate position between diseases and disability, but to add them in a separate category. Adding this group mainly reduced the contribution of background, but did not affect the contribution of the other diseases substantially. In both sexes only ‘dizziness with falling’ and ‘involuntary loss of urine’ had a high disabling impact and therefore accounted for most of the contribution of other (data available on request).

Mental health conditions are a strong predictor of disease burden and disability onset [12], [28]. Unfortunately, mental conditions were not included in the checklist of diseases in the POLS survey. To obtain an impression of the extent mental health issues contribute to the burden of disability, in an additional analysis, we included a score of 60 or lower on the RAND Mental Health Inventory (MHI-5) to the diseases, representing (light or severe) mental health problems [29]. In this analysis, 8–13% of the prevalence of disability was attributed to mental health conditions, at the expense of contributions of most other disease groups and background (Figure S1). Together with findings from previous studies, these results suggest that a substantial part of the burden of disability may be associated with mental health problems [10], [12]. However, as the MHI-5 is particularly useful for assessing mental health status of the general population and may lack validity to diagnose individual cases, the results need to be interpreted with caution. We decided to provide the results including mental health as supplementary material instead of presenting them in the main analysis.

### Comparison with previous studies

In most previous studies musculoskeletal and cardiovascular disease were reported as the most important contributors to disability [4], [5], [6], [7], [8], [9], [11]. Our study confirmed these results also for the oldest old (80+). Additionally, it was shown that the high prevalence of disability at this age is caused by a high prevalence of diseases, but even more by the high extent these diseases lead to disability at older age. This age dependence was not studied in most earlier studies. Furthermore, the current study was the first to identify PVD as an important contributor to the burden of disability, which is associated with much disability mainly by a high disabling impact.

Compared with WHOs Global Burden of Disease study, our method was substantially different [12]. Most importantly, WHOs approach uses disability weights to quantify burden of disease that are based on panel valuations and do not refer to physical (or mental) disabilities only, but represent a broader spectrum of health loss. Additionally, in this approach, each case of disease is assigned a disability weight, irrespective of individual factors such as age and sex which may affect disabling consequences of diseases, and, hence, the estimated burden of disease. In the Global Burden of Disease study, unipolar depressive disorders, alcohol use disorders, hearing loss (adult onset) and Alzheimer and other dementias were associated with most years lost due to disability in high income countries [12]. Differences in methodology and diseases included hampers further comparison with our results.

### Interpretation of results

The high prevalence of disability at older ages reflects both an increase in the presence of disabling diseases with increasing age and an increase of the disabling impact. The increasing contributions of background suggest that at older ages also frailty or age-related diseases not included in our study may have contributed [30]. This could for instance be dementia, of which the prevalence by age shows considerable resemblance with the pattern of disability attributed to background in our study [31]. Also conditions that caused persisting disability but are no longer ‘present’, e.g. falls, might have contributed [32].

Gender differences in the disabling impact of heart disease, DM, cancer, arthritis and disorder neck/arm were found. In addition to differences by gender in self-reporting behavior, differences in physiological, psychosocial and environmental factors may explain these variations. For heart disease, a higher disabling impact among females may be related to a more pronounced decline in lethality among females than males during the last decades, or to gender differences in the nature of heart disease causing better survival among disabled females than among disabled males [33], [34]. For DM, a more negative interference of the disease with protective mechanisms in the vascular wall causing thrombogenesis, and a negative influence of female gender on the effect of some cardiovascular risk factors are potential causes of the greater risk for vascular complications in females than in males [35], [36], [37]. Via cardiovascular complications, these mechanisms may also be responsible for the larger disabling impact among females. Due to differences in hormonal factors, coping styles and anxiety, females are more sensitive to pain than males [38], [39], [40]. These differences therefore may also explain the differences in the disabling impact of arthritis and disorder neck/arm.

### Implications; conclusion

This study clearly shows that diseases contribute to the burden of disability by high disabling impacts, e.g. stroke in males, by moderate disabling impacts but high prevalences, e.g. arthritis, or by both high prevalences and disabling impacts, e.g. back pain in females. Diseases that only have a high disabling impact, e.g. stroke in females, or only a high prevalence, e.g. heart disease in males, do not necessarily contribute much to the burden of disability. The current results showed that the largest contributors are musculoskeletal disorders, particularly arthritis, and CVD. For policy makers this means that the largest reductions in the burden of disability can be obtained by interventions that prevent the primary cause of disability, i.e. that prevent disease onset. Further reductions can be achieved by diminishing disabling impacts. Evidence is accumulating that effective interventions to reduce the extent diseases cause disability, such as home visit and exercise programs, are increasingly available [41], [42]. As frail elderly are particularly vulnerable to disability, this group should receive priority [43], [44]. The coming decades, the population of oldest old will increase massively, i.e. in 2050 about a quarter of the population of 50 years and older is expected to be older than 80 [45]. The large burden of disability at this age shows that reductions are urgently needed.

## Supporting Information

Appendix S1 (DOC)

Figure S1 **Prevalence of disability by cause, including ill mental health.** Abbreviations: CNSLD  =  chronic non-specific lung disease; CVD  =  cardiovascular disease; DM  =  diabetes mellitus; PVD  =  peripheral vascular disease (upper extremity excluded); MHI-5  =  RAND mental health inventory. Contributions of specific diseases to the prevalence of disability were estimated on the basis of diseases prevalence and disabling impact in the study sample from the POLS health and labor survey, the Netherlands, 2001-2007. The disabling impact represents the rate of disability from a specific cause given that the disease is present. Adding specific disability rates for the diseases present and the background rate of disability (by age and gender) gives the total disability rate for a specific exposure group. The contributions of specific diseases presented in the figure add up to the total prevalence of disability. The total prevalence for females aged> = 80 is higher than in the original analysis, which may be related with exclusion of subjects who had information missing on items for MHI-5.
(TIF)
