# Supplementary material for: Contribution of Chronic Disease to the Burden of Disability
Source: PLoS One. 2011 Sep 22;6(9):e25325. doi: 10.1371/journal.pone.0025325 (PMC3178640; doi:10.1371/journal.pone.0025325)
Supplement: Appendix S1 — (DOC) [file pone.0025325.s001.doc]

**Appendix S1. Disease groups and original questions they were compiled from.**

|  | Disease group | Original questions |
| --- | --- | --- |
|  | diabetes mellitus (DM) | 1. Do you have diabetes? |
| CVD | stroke | 2. Have you ever had stroke, cerebral bleeding or cerebral infarction? |
|  | heart disease | 4. Have you ever had a myocardial infarction?  5. Have you suffered from any severe myocardial disorder (e.g. cardiac failure or angina pectoris) |
|  | peripheral vascular disease (PVD) | 6. Have you suffered from narrow arteries in legs or abdomen during past twelve months? |
|  | cancer | 7. Have you ever had any form of cancer?  8. Have you suffered from cancer during past twelve months? (please indicate the type) |
|  | chronic non-specific lung disease (CNSLD) | 9. Have you suffered from asthma, chronic bronchitis, emphysema of lung or CNSLD during past twelve months? |
| Musculoskeletal | back pain | 10. Have you suffered from severe or persistent back disorder during past twelve months? Are you still suffering? |
|  | arthritis | 11. Have you suffered from joint degeneration during past twelve months?  12. Have you suffered from chronic joint inflammation during past twelve months? |
|  | disorder neck/arm | 13. Have you suffered from any disorder of neck or shoulder during past twelve months? Are you still suffering?  14. Have you suffered from any disorder of elbow, wrist or hand during past twelve months? Are you still suffering? |
|  | other | 15. Have you suffered from migraine or frequent severe headache during past twelve months?  16. Have you suffered from dizziness with falling during past twelve months?  17. Have you suffered from severe or persistent disorder of intestine for more than three months during past twelve months?  18. Have you suffered from involuntary loss of urine (incontinence) during past twelve months?  19. Have you suffered from psoriasis during past twelve months?  20. Have you suffered from chronic eczema during past twelve months? |
|  | RAND Mental Health inventory (MHI-5; only used in supplemental analysis) | During the past four weeks, how much of the time…  21. have you been a nervous person?  22. have you felt so down in the dumps that nothing could cheer you up?  23. have you felt calm and peaceful?  24. have you felt downhearted and blue?  25. were you a happy person? |
