# Supplementary figures and images for: Contribution of Chronic Disease to the Burden of Disability
Source: PLoS One. 2011 Sep 22;6(9):e25325. doi: 10.1371/journal.pone.0025325 (PMC3178640; doi:10.1371/journal.pone.0025325)

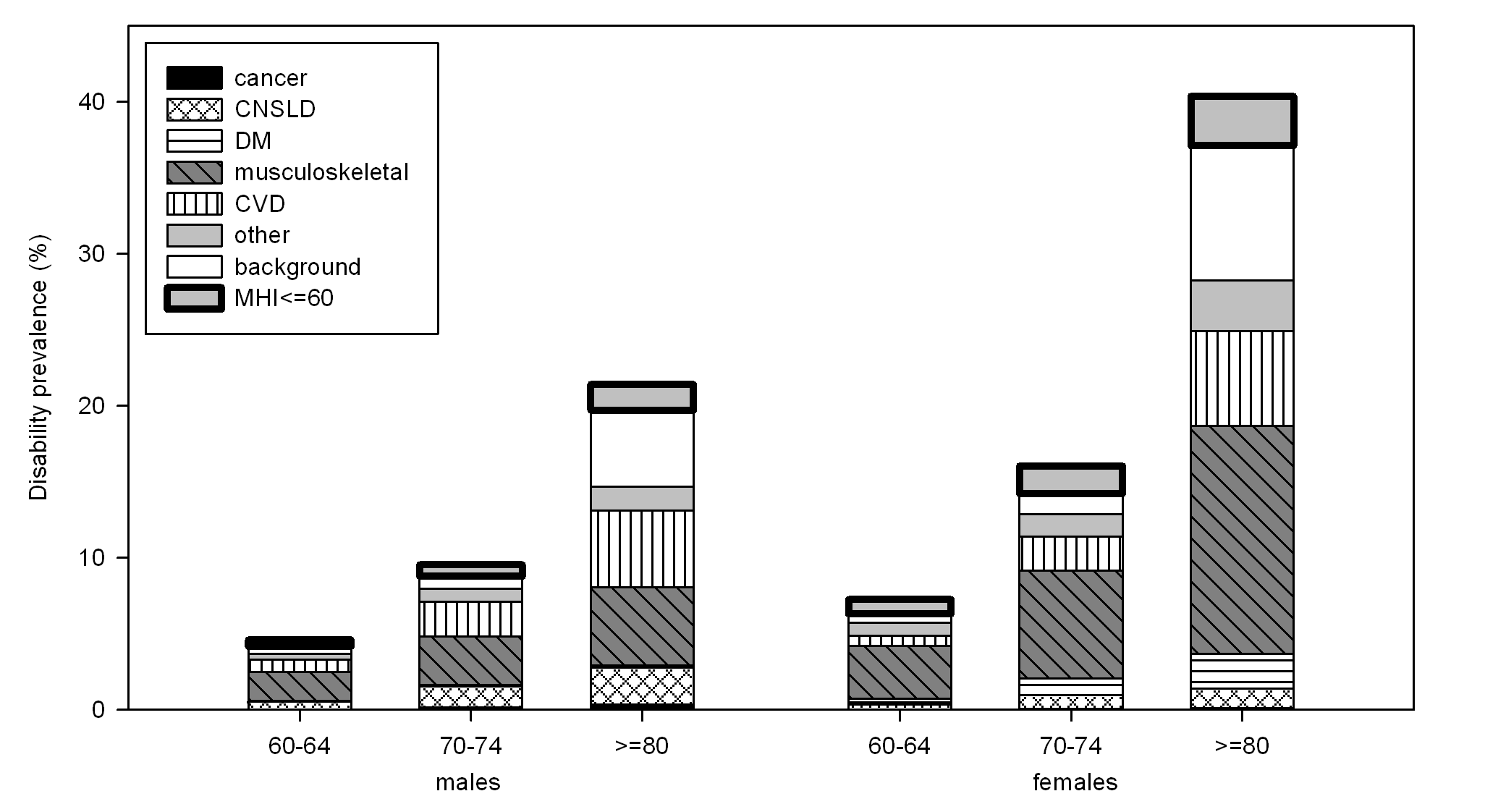

Supplement: Figure S1 — Prevalence of disability by cause, including ill mental health. Abbreviations: CNSLD = chronic non-specific lung disease; CVD = cardiovascular disease; DM = diabetes mellitus; PVD = peripheral vascular disease (upper extremity excluded); MHI-5 = RAND mental health inventory. Contributions of specific diseases to the prevalence of disability were estimated on the basis of diseases prevalence and disabling impact in the study sample from the POLS health and labor survey, the Netherlands, 2001-2007. The disabling impact represents the rate of disability from a specific cause given that the disease is present. Adding specific disability rates for the diseases present and the background rate of disability (by age and gender) gives the total disability rate for a specific exposure group. The contributions of specific diseases presented in the figure add up to the total prevalence of disability. The total prevalence for females aged> = 80 is higher than in the original analysis, which may be related with exclusion of subjects who had information missing on items for MHI-5. (TIF) [file pone.0025325.s002.tif]
